# Development and Validation of the Online Interaction Scale in Organizational Context

**DOI:** 10.3389/fpsyg.2022.884820

**Published:** 2022-04-28

**Authors:** Xin Liu, Chenhui Zhao, Zimeng Chen, Qing Wang

**Affiliations:** ^1^School of Management, Wuhan Polytechnic University, Wuhan, China; ^2^School of Business, Wuchang University of Technology, Wuhan, China; ^3^School of Business Administration, Zhongnan University of Economics and Law, Wuhan, China

**Keywords:** online interaction, human–human interaction, human–computer interaction, organizational context, measure development

## Abstract

The evolution of Web 2.0 and social networks has led to the increased use of enterprise social media platforms (ESMP), making online interactions more common in organizations. However, few studies have researched online interactions in organizational context. This study addressed this gap using two research phases: a qualitative phase and a quantitative phase. The qualitative study phase identified two dimensions of online interaction: employee–employee online interaction and employee–platform online interaction. The employee–employee online interaction assessed responsiveness and suitability. The employee–platform online interaction assessed usefulness, applicability, and ease of use. The quantitative study phase applied a proposed conceptual framework derived from the qualitative study to create and validate measures for a new online interaction scale. This was done using a systematic scale development process. Measuring online interaction can help drive future quantitative research, providing an instrumental basis for further exploring the scientific management practice elements that govern employee psychology and behavior in cyberspace.

## Introduction

As a result of the social media revolution, enterprise social media platforms (ESMP) are booming in the workplace (Yuksel and Labrecque, 2016; Leonardi and Vaast, 2017). ESMP represents a new generation of communication support information technology “with staff as the core”, which support the shift from face-to-face interaction to online interaction among employees. ESMP integrates social media elements with traditional enterprise office systems (such as e-office) or applies social technology in work contexts (such as Enterprise WeChat) (Gibbs et al., 2015). In a survey conducted by the Harrysson et al. (2016), 93% of respondents reported that their company uses at least one social technology, and 80% reported that their company uses social media for internal purposes. Many well-known companies have launched internal deployments of integrated enterprise social software services. Examples include Microsoft Yammer, Salesforce Chatter, and IBM's Connections (Choudrie and Zamani, 2016). What' more, the threat of the COVID-19 limits face-to-face interactions and offer employees opportunities to maintain interaction with organization members on ESMP. For example, in China, on February 3, 2020 (the first working day after the Spring Festival holiday), nearly 200 million people started teleworking using an ESMP (examples include Dingtalk, WeChat, and Tencent Conference). In a word, with the popularity of ESMP and the impact of the COVID-19, online interaction will become one of the most common and increasingly widespread phenomenon in organization context.

Social interactions can yield both tangible and intangible benefits, critical to any kind of exchange (Offong and Costello, 2017). In terms of online interaction, it can predict individual behavior and provide guidance for activities in a virtual environment. For example, online interaction can influence consumers' online purchasing behavior, customer loyalty and corporate performance. Yet, we have little known about online interaction in organizational context. For instance, most related, existing instruments were developed to support quantitative analysis in marketing and education. Online interaction in different contexts has different purposes, medium and subjects, and online interaction differs from face-to-face interaction in terms of space-time, symbolic cues and direction of action (Thompson, 2018). So that the existing instruments of online interaction cannot be applied in organization context. As an online interaction with unique medium and subjects, there is a need for a measure designed to evaluate.

In addition, as a medium for online interaction, ESMP has raised the interest of many management scholars. Recent studies have found that ESMP enables more efficient, transparent, and convenient communication, and that it promotes knowledge sharing and task performance (Cai et al., 2018; Fu et al., 2019; Sun et al., 2020; Luqman et al., 2021). The technical characteristics of ESMP promote a prosperous work environment for newcomers (Sun et al., 2019; Cai et al., 2020), effectively reduce network idling behavior (Nivedhitha and Manzoor, 2020), and establish social bonding in the workplace (Ollier-Malaterre, 2013; Shang and Sun, 2021). Although, technical characteristics of ESMP can support employee interact without the restrictions of time and space, increasing technical characteristics of media does not ensure stronger perceptions of interaction. Therefore, in terms of the question of “whether online interaction in an organization is subjective or objective,” we need more in-depth research. Furthermore, the researchers who examined online interaction in organization from the perspective of perception only focused on online interaction among employees (Huang et al., 2017), while the human–computer interaction in organization hasn't be concerned.

The above highlights the need to view online interactions in organizational contexts, which is the goal of this paper. Specifically, we applied qualitative research methods to better understand the meaning of online interaction from the perspective of perception. Based on the qualitative results, we applied quantitative methods to develop a multidimensional scale that assesses online interaction in the organizational context.

## Literature Review

### Concept and Measure of Online Interaction

We synthesized arguments from the literature to provide a theoretical basis for describing online interaction in organizational contexts. Most scholars have defined concepts related to online interaction from three perspectives. Table 1 shows concept and measures for researching online interaction.

**Table 1:** Concept and measures for researching online interaction.

**Perspective**	**Dimensions**	**References**
Structure	Active control, two-way communication, synchronicity	Liu and Shrum, 2002; Liu, 2003
Content	Sociability, functionality, reactivity and initiative; information-oriented interaction, task-oriented interaction and relations-oriented interaction.	Nambisan and Baron, 2007; Köhler et al., 2011
Perception	Operability; ease of use; perceived control, perceived response, perceived personalization	Wu, 2006; Zhao and Lu, 2010

First, some scholars have proposed that interaction should be measured using the structure of the media for interacting. The dimension of online interaction include active control, two-way communication and synchronicity, which mainly concerns technical features of media (Liu and Shrum, 2002). A key problem of measuring technical features is that objective technical characteristics can facilitate interaction, but do not guarantee that people actually use these technologies to interact. Therefore, online interaction should have some meaningful social and psychological relevance (Bucy, 2004), and test online interaction from the perspective of subject of interaction rather than the medium of interaction.

Second, some studies have focused on the content of interactions. For example, Köhler et al. (2011) used coding to extract conversational content between customers and online agents, obtaining interactive content about the characteristics of sociability, functionality, responsiveness, and initiative. In addition, organizational researchers have also considered the content of interactions to be important (Bonner, 2010).

Third, the broad view focuses on the experience and perception of the user during the process of interaction. For example, Wu (2006) focused on perceptual control, perceptual response, and perceptual personalization to measure online interactions between consumers and shopping sites.

Although, there's no a universal model describing the dimensions of online interaction, some researches certain that, compared with objective interactions studied from a structural perspective, subjective interactions studied using the perspectives of experience and perception, can effectively predict individual attitudes and behaviors (Mcmillan and Hwang, 2013; Yang and Shen, 2017).

### The Types of Online Interaction

#### Online Interaction Mediated by Different Social Media

Thompson (2018) focused on “Mediated online interaction,” proposing that communication media is embedded in many different types of social organizations. For example, enterprises develop “We Media,” which is used to promote corporate image, products, and services, and expand corporate influence; academic researchers search academic resources using virtual academic exchange platforms; job seekers use platforms of recruitment (e.g., Job.com, LinkedIn) to search for information about job. In addition, individuals establish personal social media accounts to establish connections with other groups, memberships, or organizations in society, obtaining information resources and even earning money (Yuksel and Labrecque, 2016). In summary, different social media embedded in social organizations serve different audiences and purposes, which means there are specific objects of online interaction in different social media tools. In organization context, ESMP is a highly used medium, and serves the work and communication of the organization members.

#### Online Interaction Between Different Subjects

Based on different interaction subjects, online interaction can be divided into human–human interaction and human–computer interaction (Hoffman and Novak, 1996). There are different subjects in different situations, forming different types of human–human interactions. For example, in a marketing context, interactions include customers, enterprises and employees, which form customer–customer online interactions (Bonner, 2010; Yoo et al., 2012), enterprise–customer online interactions, and employee–customer online interactions (Wu, 2006). In the field of education, there are learner–content online interactions, learner–instructor online interactions, and learner–learner online interactions (Alghasab et al., 2019). As ICT has developed, the subject of human–computer interaction is no longer limited to a computer. Instead, they include a variety of electronic devices, diversified applications, and social media (Kovacova and Lazaroiu, 2021; Pelau et al., 2021).

### Our Conceptualization of Online Interaction in Organization

#### The Need for a Conceptualization of Online Interaction in Organizations

Some researchers have made strides in understanding online interaction (Oh and Sundar, 2015; Vazquez et al., 2017; Yang and Shen, 2017); however, some topics deserve further attention. First, few measurement constructs have been developed using a formal scale development process to assess online interaction (Liu, 2003). Some previous studies mostly used simple descriptive analysis to evaluate and classify the types and dimensions of online interaction, and do not place the online interaction in a specific context during the process of scale development, which results in an inability to effectively establish the validity and reliability of the scales. Second, some past studies on online interaction mainly occurred in marketing and education contexts. However, the situational dependencies of individual studies limit the application of existing research to the organization. As such, the literature is generally silent on the phenomenon of online interaction in the workplace.

Given the complexities of online interaction and the lack of a conceptual and empirical understanding about online interaction in organizational contexts, we began the conceptualization process by clarifying the types of online interactions in organizational contexts, the perspective of scale development, and the complexities of the construct dimensions.

#### The Types of Online Interactions in Organizational Situation

A systematic review of the connotations, dimensions, and types of online interaction reveals that different media and subjects and objects constitute different types of online interaction. In the organizational context, ESMP is a frequently used type of media for online interaction, and employees are the subject of online interaction. As important members of the organization, employees are the main body of online interaction and are the main subjects serviced by ESMP. Thus, according to Hoffman and Novak (1996), we posit there are two types of online interaction in organizational contexts: employee–employee online interaction and employee–platform online interaction. Employee–employee online interaction is associated with human–human interaction, which refers to the online interaction between employees and other members of the organization. Employee–platform online interaction is associated with human–computer interaction, which refers to the interaction between employees and ESMP.

#### The Perspective of Scale Development

Previous research experience has shown that the definition and scale of online interaction differs between different perspectives. The different perspectives all focus on a same problem: whether interaction is subjective or objective, and how much interaction is in the eyes of the beholder. Liu and Shrum (2002) noted the difference between the structure of interaction and perception of interaction. Structure refers to the hard-wired opportunity provided in the interactive process. Perception refers to the perception of participants in the interaction, reflecting the subjective interactivity. Scholars have found that increasing the interactive function of media does not ensure stronger interactive perception (Sohn, 2011). Subjective interactivity is significantly higher than objective interaction with respect to the prediction effect of individual behavior (Mcmillan and Hwang, 2013; Yang and Shen, 2017). Furthermore, perceptual variables support empirical measurements and help promote the theoretical development of this concept, and Bucy (2004) confirm that interaction from the perspective of perception conforms to people's customary understanding of interactivity.

In this study, however, the perspectives of structure and content do not highlight the inherent meaning of online interaction in an organizational context. First, the perspectives of structure emphasize technical characteristics, and do not consider the employee's psychology and behavior. Second, although the content of the interaction is important (Bonner, 2010), it is more important to recognize that information and symbolic content merely be transmitted by media, which remain essentially unchanged. As such, developing the scale of online interaction in the organizational context from a content perspective does not effectively distinguish the differences between online interaction and face-to-face interaction, and does not consider the interaction between employee and the ESMP.

When considering the question of “how to measure online interaction in an organization and whether it is subjective or objective,” we hypothesize that the experience and perception gained by employees during the process of interaction is most important. The online interaction in the organization is defined as the perception of employees who interact with other members in the organization or transfer information on the ESMP.

## Qualitative Study Phase

To capture occurrences of online interaction in an organization, we conducted a series of qualitative studies with people across many organizations with ESMP to explore the structure and dimension of the target construct. Grounded theory is a kind of qualitative research method, which requires researchers to summarize experience directly from actual observation or original data, and then rise to systematic theory (Corbin and Strauss, 1990). Based on the grounded theory method, we referred to research steps of Fassinger (2005) to refine the dimensions of online interaction in organizational context through data collection, data coding and concept extraction.

### Data Collection

We invited employees using our team members' address book. Online interaction involves a key element, the use of an ESMP; as such, candidate participants were screened to identify people with experience in using ESMP to participate as interviewees. After this process, a total of 25 interviews were conducted, with four teachers, seven engineers, two accountants, two human resource (HR) specialists, three salesmen, one lawyer, two programmers, one civil servant, one risk manager, and two bankers. Their mean work experience was 4.28 years, and 48% of the study subjects were male.

Interviews were conducted in three ways: face-to-face interview, telephone interview, and video interview. Each interview was recorded and archived. To inform interviewees about the purpose and confidentiality of the study, each interviewer took 1–5 min to briefly explain the purpose of the research and committed to keeping the personal information confidential before the official interview. This meant that no third party, including the employer, would have access to their personal information, and data collected would be used only for academic purposes. Interviewers were instructed to explain the interview questions clearly to help the interviewees accurately understand the questions; interviewers also encouraged interviewees to voice their thoughts by giving examples, if they were less talkative.

Each interview was semi-structured, lasted about 30 min, and included questions about interviewee's perceptions and experiences during the process of online interaction in their organizations. Specifically, we instructed the participants to describe what they perceive and experience when they communicate with other employees on the ESMP; what aspects they pay more attention to when they use the ESMP to communicate with others; and whether they have encountered any problems (and if yes, what specific problems) when using the ESMP.

### Coding Process

Before coding, we processed the interview data to extract statements or fragments relevant to the study. The coding process involved three stages: open coding, axial coding, and selective coding (Fassinger, 2005).

#### Open Coding

Open coding means conceptualizing and discovering categories through continuous refinement, reduction, and recombination of raw materials. There were four steps and two coders involved in the open coding stage. The coders were Ph.D. students majoring in human resources management.

The first step was labeling. Two coders independently labeled the main information from the original sentence or paragraph (Corbin and Strauss, 1990). This original sentence or paragraph should be relevant to employee perception and experience during the process of online interaction between employee and employee, employee and platform. Then, according to the rule of maximum probability, they combined all the labels, generating 187 tags.

The second step was conceptualization. In order to improve the reliability of coding results, the two coders also worked independently to abstracted and classified the 187 tags. For example, the statement, “It may have been several days since I saw the message on WeChat,” can be conceptualized as “Not checking information in a timely way.” Then, the two coders compared the results together and pick out the same or similar conceptual. The two coders ultimately logged the same or similar conceptual encoding results for 136 of the 187 tags. The coding results are tested according to the mutual agreement and reliability formula proposed by Holsti (1969). We calculated a mutual agreement of *K* = 0.719 and result reliability of *R* = 0.836. The coding consistency was higher than the required reliability standard of 0.80 (Latham and Saari, 1984).


(1) $$\begin{array}{l} K=\frac{2\text{M}}{\text{T}1+\text{T}2} \end{array}$$

(2) $$\begin{array}{l} R=\frac{\text{N }*\text{ K}}{1+\left(\text{N}-1\right)\;*\text{ K}} \end{array}$$

In this expression, K is the coders' average degree of mutual agreement; T1 and T2 are the number of codes by coders, respectively; M is the number of identical codes; R is the result reliability of coding; and N is the number of coders.

The third step was to delete duplicate and redundant codes. Some codes in the 136 codes appeared many times. For example, “ambiguity in text communication” appeared six times; “respond to messages promptly” appeared six times; and “convenience of the platform” appeared fourteen times. A total of 39 codes were left after deleting duplicate codes from the 136 codes. To improve coding reliability, we further eliminated the codes appearing less than two times from the 39 codes. A final set of 24 codes were identified during this step.

The fourth step was categorization, to identify and group synonymous codes under a unified concept. For example, “not given feedback in time” and “feedback information in time” were grouped using a unified concept called, “timeliness of information feedback.” As a second example, the phrases “Receive messages that are not work-related” and “Invalid feedback content” were grouped using a unified concept called, “effect of communication.” A third example was that “appropriate tone of communication” and “appropriate time of communication” were grouped under the unified concept “communication etiquette.” The 24 codes were therefore reduced to 12 initial concepts.

#### Axial Coding

In the axial coding stage, research was applied to identify and establish the different relationships among the categories (Hamlin and Patel, 2020). This resulted in five major categories from the original 12 initial categories: responsiveness (effect of communication, timeliness of information feedback), suitability (etiquette of communication, use of appropriate social media, use of the appropriate information carrier), usefulness (the function of the platform, promoting work efficiency, promoting interaction between employees), applicability (degree of matching between platform confidentiality and work requirements, the degree of matching between the platform's foundation capability and work requirements), and ease of use (platform integration, convenience of platform operation).

#### Elective Coding

The elective coding stage involved grouping the core categories, by comparing and analyzing the five main categories against the existing research dimensions of online interaction (Corbin and Strauss, 1990). For example, “responsiveness” and “suitability” involve the online interaction between the employee and employee; “usefulness,” “applicability,” and “ease of use” involve the interaction between employees and the ESMP. This summarized the five main categories into two core dimensions: “online interaction between employees” and online interaction between employee and the ESMP.”

### Coding Results

The 25 interviews and three stage coding process above revealed that employee–employee online interaction includes two sub-dimensions: responsiveness and suitability. Employee–online interaction includes three sub-dimensions: usefulness, applicability, and ease of use. Table 2 shows representative quotes from interviewees for the coded items.

**Table 2:** Representative quotes from interviewees for the coded items.

**Variable**	**Dimension**	**Items**	**Representative quotes**
Employee–employee online interaction	Responsiveness	Timeliness of information feedback	We asked him to give us the feedback of this form, and they did not give timely feedback. We have to @ him in the Wetchat's group. Then he may give it to you.
		Effect of communication	After reading the text from my colleague, I don't know what he wants to ask me and what he needs me to do.
	Suitability	Use of the appropriate information carrier	Wechat is mainly used for typing, although it also has voice function. Our colleagues in functional departments do not like to answer the phone, especially after the company introduced Wechat. They are used to seeing the text you sent over, and they are too lazy to listen to the voice you sent.
		Etiquette of communication	My tone of online communication is still different from my usual tone, I usually add a mood word at the end of each sentence.
		Use of appropriate social media	Generally, I will not check my work wechat when I go home from work. For several times, when I log in wechat on my computer the next day at work, many unread messages will not be displayed. As a result, the work content sent by many colleagues cannot be checked, which is easy to cause misunderstanding and delay work.
Employee–platform online interaction	Usefulness	The function of the platform	The platform has narrowed the distance between departments and overcome regional communication barriers. We use Wechat 99% of the time at work. Wechat plays a great role in the work, supporting the sending of text, pictures, videos, voice messages, all kinds of documents.
		Promote work efficiency	
		Promote interaction between employees	The platform is highly targeted to our daily work, and can solve the communication and exchange between colleagues.
	Applicability	Degree of matching between platform confidentiality and work requirements	Our company has developed own platform for internal communication. The platform is used on the Intranet with high confidentiality.
		The degree of matching between the platform's foundation capability and work requirements	Documents on Wechat will become invalid if they cannot be accepted in time, and the expiration of documents will cause inconvenience for work.
	Ease of use	Convenience of platform operation	This platform is required by the company, mainly for the confidentiality of enterprise information. I feel that the user experience is not very good. Because the platform is not very stable without the Intranet. I often fail to log in or get disconnected easily when I am on a business trip.
		Platform integration	Different companies have different systems. Generally, one kind of EMSP can't meet all our work needs. The using of Wechat and Dingtalk account for the majority in our daily work, and they are mainly used in work communication.

#### Employee–Employee Online Interaction: Responsiveness and Suitability

When considering the first sub-dimension of the employee–employee online interaction, responsiveness, there were two elements: the speed of response and the effect of feedback. These results echo previous research, which emphasized that interaction should allow the two-way flow of information; the information exchanged in order should be closely related; and the information exchange should be carried out quickly. In other words, when one communicator sends a message, the other party should also quickly make a sound (Wu, 2006; Van Noort et al., 2012). Our study also extracted a second sub-dimension, suitability, in employee–employee online interaction. Suitability refers that the employee perceives the etiquette of communication among colleagues, the degree of matching between the information carrier and social media used by colleagues with the habits and work characteristics of the employee. In an organizational context, suitability during the online interaction is significant when delivering information to others. A similar concept in marketing is known as personalization. Personalization means that shopping platforms provide services tailored to customers' characteristics, needs, or habits for marketing purposes (Mulvenna et al., 2000).

#### Employee–Platform Online Interaction: Usefulness, Applicability, and Ease of Use

When considering the employee–platform online interaction, this study found that employees focus on the usefulness and ease of use of the ESMP, and to the degree of matching between the platform's capabilities and the employee's work requirements. This second element is called applicability. Past studies measuring human–computer interaction have mainly focused on usefulness and ease of use. However, anecdotal evidence provided by the semi-structured interviews indicated that perceived usefulness and ease of use were insufficient on their own to capture employee perceptions with respect to online interactions in the workplace.

With the increased complexity of work, employees are demanding more from ESMP during online interactions; in the information age, knowledge has exploded beyond human control. Employees need to share resources, obtain information, and store resources during the process of online interaction, and ESMP need to meet their needs. Researchers have acknowledged that technologies need to match individual needs, and the degree of matching between the two influence customers' shopping intentions and user behavior (Vijayasarathy, 2004; Wu and Wang, 2005). Our interviews also found that due to the needs of work, employees have higher demands for information transmission, information storage, and platform confidentiality. To remain consistent with this, we introduce “applicability” to reflect employee perceptions when they interact with the platform.

## Quantitative Study Phase

After exploring the structure and dimensions of the target construct, we followed Churchill (1979) recommendations for scale development, creating an online interaction scale through item generation, scale refinement, and validation (Mawritz et al., 2020). To be specific, the quantitative phase of this study included three studies. Study 1 generated an initial pool of items, which were then reduced based on redundancies observed by the authors and based on advice from academics with expertise in scale development (Zheng et al., 2015). Study 2 built on the first study to further establish content validity, and further refine and reduce the items through exploratory factor analysis (EFA). Study 3 further refined the scale using confirmatory factor analysis (CFA) and tested the convergent and discriminant validity of the scale.

### Study 1: The Initial Item Generation

For Study 1, we created an initial pool of items combining the 24 items developed in the qualitative research and 43 items from existing scales, such as four items from a responsiveness scale (Yin, 2002), fourteen items from a perceived ease of use scale and perceived usefulness scale (Ahn et al., 2004), and three items from a compatibility scale (Wu and Wang, 2005).

The following three principles were observed in the selection of the scale items, to ensure the scale's reliability and validity: (1) If the item developed in the qualitative research was similar to an item in a mature scale, the item from the mature scale was selected; (2) If the measurement content of the mature scale item is inconsistent with an item developed in the qualitative research, the item developed in the qualitative research was selected; and (3) If there was no mature scale for reference, the item developed in the qualitative research was used. This comparison and screening process yielded 21 initial scale items.

The expert evaluation method helps increase content validity (Hinkin and Schriesheim, 1989). As such, we invited five PhD students in management to review the 21 items, assessing them against the dimension definitions. First, we explained the meaning of each dimension to five PhD students, using illustrative examples. The five PhD students categorized the 21 items into the most appropriate dimensions, and the results of their classification were then integrated. The integration results identified one item that did not belong to any dimension and was removed by more than 3 experts; the item was: “The ESMP currently in use is relatively concentrated.” To further improve content validity, we invited three different managers to revise and proofread the statements associated with the remaining 20 items. Table 3 shows the final scale of online interaction in organizations.

**Table 3:** Initial measurement of online interaction in the organization context.

**Number**	**Items**	**References**
Q1	On ESMP, colleagues reply me information quickly.	Interview, Yin (2002); Wu (2006); Mcmillan and Hwang (2013)
Q2	On ESMP, colleagues offered information relevant to the questions I asked.	Interview, Yin (2002); Wu (2006); Mcmillan and Hwang (2013)
Q3	On ESMP, colleagues don't send information that is not work-related.	Interview, Yin (2002); Wu (2006); Mcmillan and Hwang (2013)
Q4	On ESMP, I think my communication with colleagues is usually effective.	Interview
Q5	On ESMP, colleagues can choose the appropriate time to communicate with me.	Interview
Q6	On ESMP, colleagues can use the appropriate tone to communicate with me.	Interview
Q7	On ESMP, colleagues can choose the appropriate carrier of information to communicate with me.	Interview
Q8	In order to adapt to the characteristics of my work, my colleagues will cooperate with me to use a variety of ESMP.	Interview
Q9	The features of ESMP are all useful to me.	Interview, Gefen et al. (2003); Ahn et al. (2004)
Q10	The ESMP helps me get useful information.	Interview, Gefen et al. (2003); Ahn et al. (2004)
Q11	The ESMP facilitate interaction between employees.	Interviews
Q12	The ESMP improves work efficiency.	Interview, Gefen et al. (2003); Ahn et al. (2004)
Q13	The file storage capacity of ESMP currently in use meets my needs of working.	Interview
Q14	The file transfer capability of ESMP currently in use meets my needs of working.	Interview
Q15	The ESMP currently in use is confidentiality enough to meet my needs of working.	Interview
Q16	The ESMP currently in use provides all the functionality I need to do my work.	Interview
Q17	It is easy for me to become skillful at using the ESMP.	Interview, Ahn et al. (2004); Wu (2006)
Q18	It is easy for me to access fluently to the ESMP.	Interview, Ahn et al. (2004); Wu (2006)
Q19	The ESMP currently in use incorporates a variety of functions.	Interview
Q20	I can use the various functions of ESMP without restriction.	Interview

### Study 2: The Reduction of the Initial Items

In study 2, we establish content validity by testing the scale's dimensionality and further reducing the item pool. To determine if items were grouped within intended subscales, we performed an EFA, using principal axis factoring with oblique promax rotation (Costello and Osborne, 2005; King and Bryant, 2017).

#### Participants and Procedure

Participants for Study 2 were recruited through the researchers' personal contacts, such as relatives, friends, and classmates. The study questionnaire was also posted on a third-party questionnaire collection platform. A total of 138 electronic questionnaires were collected; questionnaires with the same answer for 10 consecutive questions or an unusually regular distribution of answers were eliminated. After this screening step, 103 valid questionnaires remained, with an effective response rate of 74.64%.

The scale at this step had 20 measurement items. As such, a sample size of 103 respondents was determined to meet the statistical requirement of a sample that was 5–10 times the number of items. In terms of demographics, 58.3% of respondents were male and 41.7% of participants were female; 9.7% of participants were under 25 years old; 61.2% of participants were between 26 and 30 years old; and 29.1% of participants were over 30 years old. Participants responded to the survey items using a 5-point Likert scale (1 = not entirely agree; 5 = entirely agree).

#### Results

Before conducting EFA, however, we needed to perform a Bartlett's test to determine whether the sample was suitable for EFA (Chan et al., 2021). The Bartlett's test found that the correlations, when taken collectively, were significant at the 0.001 level. This demonstrated that the data were suitable for EFA.

The following three criteria were used to determine whether the item loaded onto an underlying factor: (a) the item had a factor loading of 0.60 or better on one factor; (b) the item had a loading of <0.40 on the second factor; and (c) the cross-loading differential across the two factors was <0.25 (Costello and Osborne, 2005; Giordano et al., 2019). Q8 and Q6 were deleted according to the above criteria. This is because Q8 loaded 0.59 on Factor 2, while it loaded 0.358 on Factor 5; and Q16 loaded 0.430 on Factor 2 and 0.467 on Factor 5. The other items had a single dominant factor loading in the rotated solution, making factor inclusion relatively straightforward. Table 4 shows that the five factors explained 78.313% of the total variance. The online interaction scale demonstrated a high level of internal consistency (α = 0.917).

**Table 4:** Item loading values from exploratory factor analysis.

**No**.	**Factor**				
	**Factor 1**	**Factor 2**	**Factor 3**	**Factor 4**	**Factor 5**
Q1	0.209	**0.767**	0.183	0.079	0.256
Q2	0.198	**0.904**	0.116	0.142	0.013
Q3	0.21	**0.908**	0.158	0.112	0.133
Q4	0.329	**0.515**	0.337	0.202	0.184
Q5	0.164	0.139	**0.896**	0.072	0.084
Q6	0.194	0.154	**0.908**	0.021	0.174
Q7	0.31	0.23	**0.813**	0.042	0.077
Q9	**0.767**	0.14	0.235	0.182	0.198
Q10	**0.809**	0.169	0.277	0.125	0.164
Q11	**0.762**	0.25	0.196	0.089	0.226
Q12	**0.800**	0.334	0.127	0.092	0.153
Q13	0.282	0.126	0.188	0.201	**0.775**
Q14	0.304	0.092	0.092	0.293	**0.711**
Q15	0.085	0.191	0.114	0.204	**0.798**
Q17	0.195	0.06	0.149	**0.876**	0.136
Q18	0.195	0.217	−0.048	**0.657**	0.394
Q19	−0.099	0.046	0.009	**0.707**	0.181
Q20	0.253	0.159	0.047	**0.808**	0.072
Cumulative percentage of variance				78.313
Cronbach's αs				0.917

*Values in bold indicate maximum factor load capacity for each item*.

The first dimension, responsiveness, included four items: “On ESMP, colleagues reply to me with information quickly;” “On ESMP, colleagues offer information relevant to the questions I asked;” “On ESMP, colleagues don't send non-work-related information;” and “On ESMP, I think my communication with colleagues is usually effective.”

The second dimension, suitability, included three items, including “On ESMP, colleagues can choose the appropriate time to communicate with me;” “On ESMP, colleagues can use the appropriate tone to communicate with me;” and “On ESMP, colleagues can choose the appropriate mode, or carrier of information, to communicate with me.”

The third dimensions, usefulness, included four items related to the perception of ESMP usefulness: “The features of ESMP are all useful to me;” “The ESMP helps me get useful information;” “The ESMP facilitates interaction between employees;” and “The ESMP improves work efficiency.”

The fourth dimension, perceived ease of use when employees use the ESMP, included four items: “It is easy for me to become skilled at using the ESMP;” “It is easy for me to access fluently to the ESMP;” “The ESMP currently in use incorporates a variety of functions;” and “I can use the various functions of ESMP without restriction.”

The fifth dimension, applicability, included three items: “The file storage capacity of ESMP currently in use meets my working needs;” “The file transfer capability of ESMP currently in use meets my working needs;” and “The ESMP currently in use is secure enough to meet my working needs.”

Based on the EFA, we concluded that online interaction has five dimensions: responsiveness, suitability, usefulness, applicability, and ease of use. This confirmed our formulated proposal of online interaction in the qualitative study.

### Study 3: Confirmatory Factor Analysis

To determine if the factor analytic structure of the scale in Study 2 was replicable, and to further examine evidence for validity, we applied CFA and competitive model analysis techniques.

#### Participants and Procedure

With support from the human resources department, we were able to access a large state-owned power enterprise in China with more than 40,000 employees to administer the questionnaire survey. Consistent with our research requirements, the organization stratified its employees to allow random sampling in accordance with the proportion of the sector structure. Five hundred and seventy-eight employees were chosen from functional departments and production departments; these departments included the human resources department, labor union, sales department, administration of power supply, production technology department, and other subsidiaries. The questionnaire distribution lasted for 1 month. A total of 337 valid questionnaires were obtained, with an effective rate of 78.9%.

Among the 337 participants, 219 were male and 118 were female. In terms of sample age, 13 participants were under 30 years old, accounting for 3.9%; 16 persons were aged 31–40, accounting for 4.7%; 209 participants were aged 41–50 years old, accounting for 62.0%; 99 participants were over 50 years old, accounting for 29.4%. In terms of educational background, 188 participants had an associate degree, accounting for 55.8%; 126 participants had a bachelor's degree, accounting for 37.4%; and 23 participants had a master's degree or above, accounting for 6.8% of the participants. A total of 13.6% participants worked <5 years in the current enterprise.

#### Result

**Figure 1:**
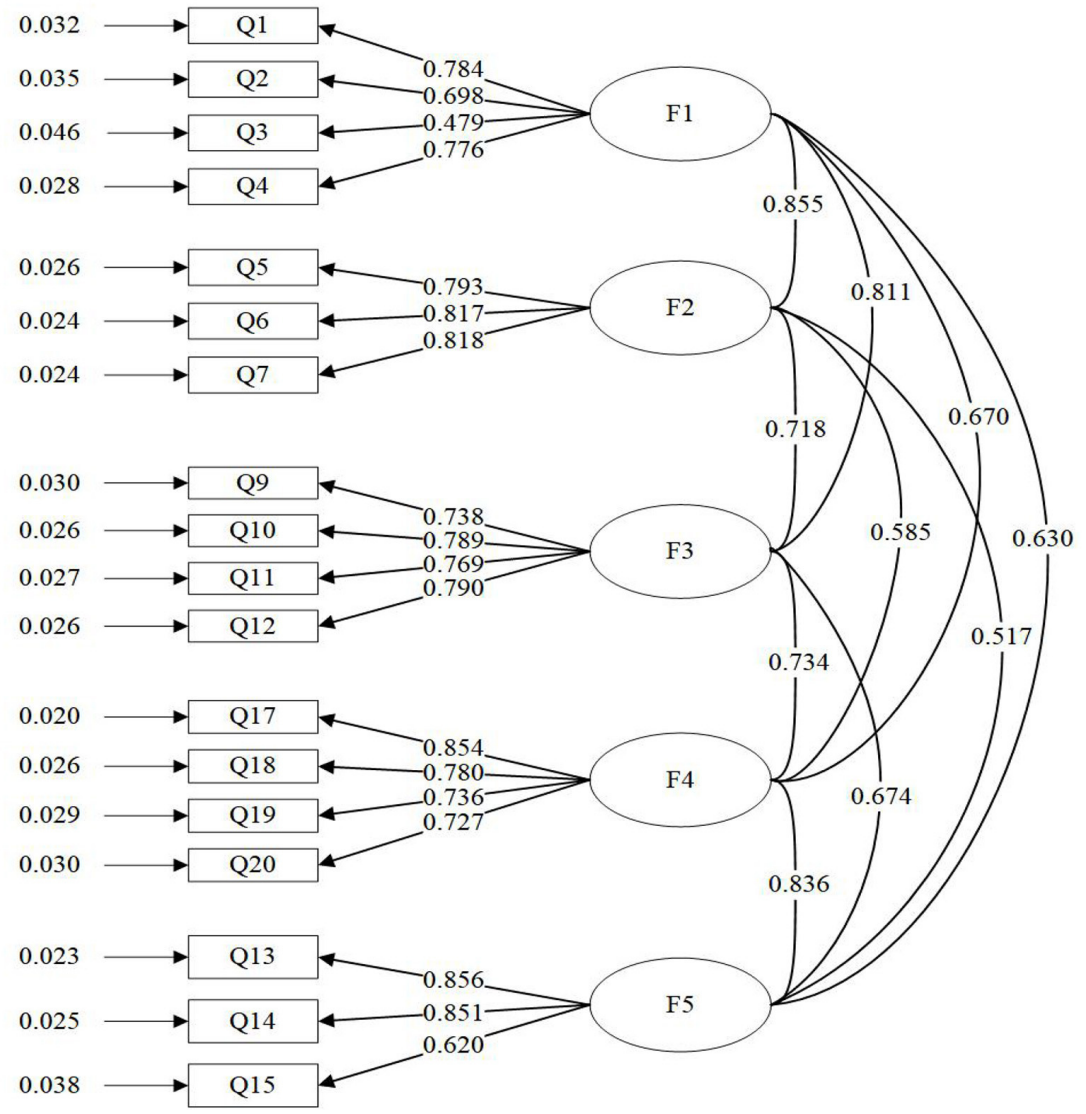
Estimations of the standardized path coefficient of the confirmatory factor model.

**Figure 2:**
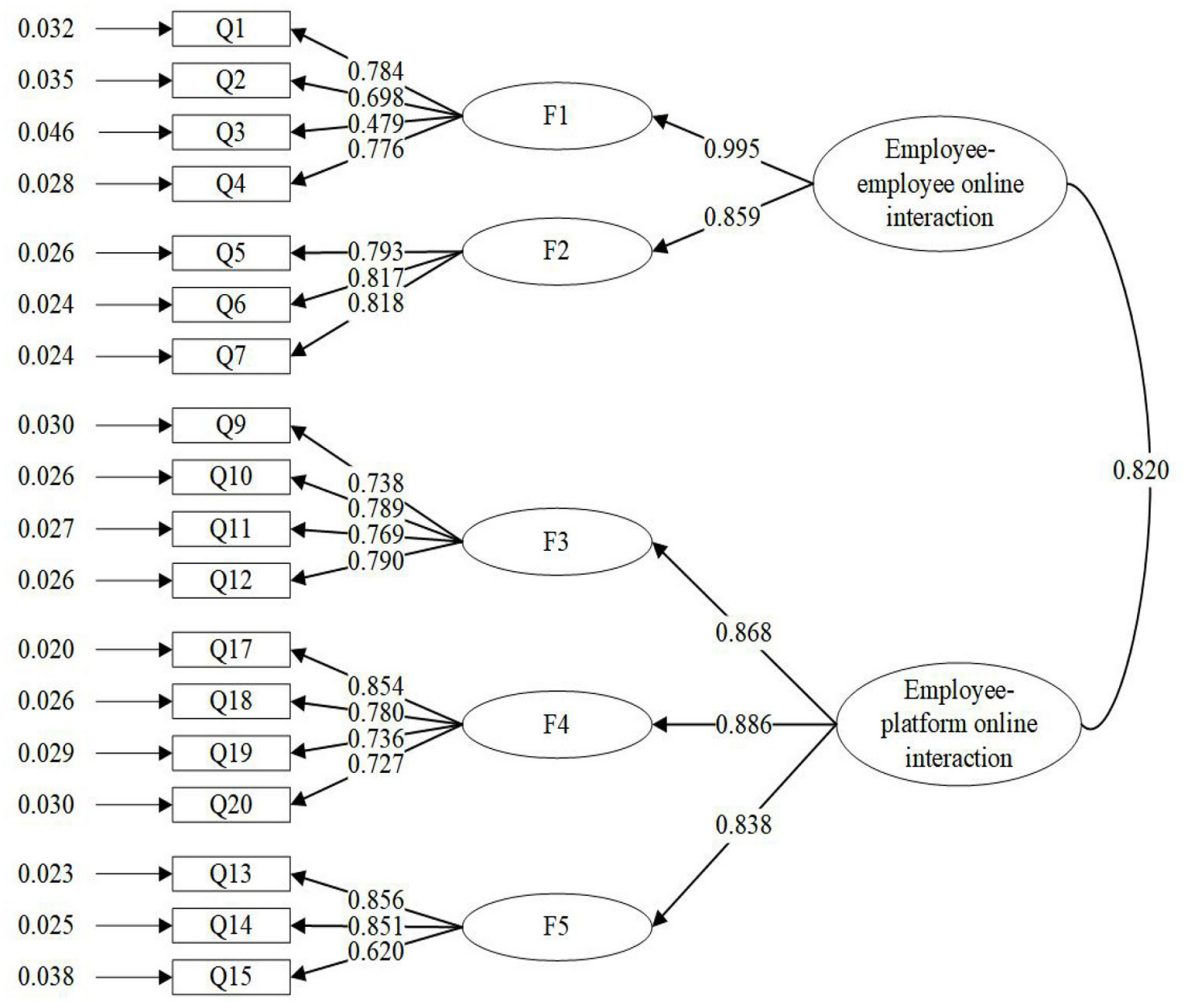
Estimations of the standardized path coefficient of the final confirmatory factor model.

We analyzed the CFA using Mplus7.4, a statistical software package. Each item in the five first-order model of online interaction had a relatively high load on the corresponding potential variables, and the standardized load distribution of each item was between 0.465 and 0.843. This indicated that each factor has a high explanatory rate on the corresponding potential variables (Williams et al., 2010). Figure 1 shows that in the first-order factor model, the correlation coefficient between factors was as low as 0.517 and as high as 0.855. This laid the foundation for a second-order factor model analysis (Peterson, 2014). To determine if a second-order factor model for the online interaction was appropriate, we combined the first-order factor model into a second-order factor model, as shown in Figure 2.

In addition, to determine which measurement model fit the data best, we compared four models: one second-order model (Model 1) and three first-order models (Models 2 to 4). The results are shown in Table 5. Model 1 assessed the merger of responsiveness and suitability as one factor (Employee–Employee online interaction), and the merger of usefulness, applicability, and ease of use as one factor (Employee–ESMP online interaction). These, in turn, load on a second-order factor (online interaction). Model 2 assessed how the 18 observable items load on the five first-order factors (responsiveness, suitability, usefulness, applicability and ease of use). Model 3 assessed how the 18 items are accounted for by the single first-order factor, online interaction. Model 4 assessed how the 7 items of responsiveness and suitability are accounted for by a single first-order factor (employee–employee online interaction), and how the 11 items of usefulness, applicability, and ease of use are accounted for by the single first-order factor of employee–platform online interaction.

**Table 5:** Major fitting degree indices of online interaction.

**Model number**	**Model**	**χ^2^**	**df**	**χ^2^/df**	**SRMR**	**TLI**	**CFI**	**RMSEA (90% CI)**
1	Second-order model	385.636	129	2.989	0.052	0.911	0.925	0.077 (0.068, 0.086)
2	Five-factor model	336.956	125	2.696	0.042	0.924	0.938	0.071 (0.062, 0.080)
3	Signal-factor model	944.122	135	6.993	0.079	0.730	0.762	0.133 (0.125, 0.141)
4	Double-factor model	673.218	134	5.024	0.067	0.819	0.842	0.109 (0.101, 0.118)

*SRMR, standardized root mean square residual; TLI, tucker-lewis index; CFI, comparative fit index; RMSEA, rood mean square error of approximation*.

Fit indexes (Table 4) showed that Model 1 was the best fit compared to other models (χ^2^ = 385.636, df = 132, TLI = 0.911, CFI = 0.925, RMSEA = 0.077); Model 2 was the next most effective fit (χ^2^ = 336.956, df = 125, TLI = 0.924, CFI = 0.938, RMSEA = 0.071). According to the principle of parsimony, the second-order Model 1 was selected as the best model. Figure 2 shows that the lowest load of the first-order factor on the second-order factor was 0.838, indicating that about 60% (0.838^*^0.838) of the variation of this factor was explained by the second-order factor, supporting the existence of the second-order factor (Hou et al., 2004).

## Discussion

There are many ways to conceptualize online interaction; however, there remains a lack of clarity with respect to its structure and measures in an organizational context. To fill this theoretical gap, we applied both qualitative and quantitative methods to identify the theoretical structure of online interaction. In the qualitative phase, we explored possible dimensions of online interaction in organization context by conducting semi-structured interviews. In the quantitative phase, items were proposed by combining a literature review and the consumer interviews in study 1; items preliminarily examined the items and reduce the initial items by EFA in study 2; tested the validity of the scale by CFA in study 3.

The results show that, online interaction in organizations can be assessed in terms of the responsiveness and suitability of the employee–employee online interaction; the employee–platform online interaction can be assessed in terms of the usefulness, applicability and ease of use. Using a series of quantitative studies, we developed an 18-item online interaction scale and established its reliability and validity. This study makes an important contribution to online interaction studies in organizational contexts, and plays a complementary role in enriching and extending existing online interaction theories and empirical research.

## Theoretical Contributions

By conceptualizing online interaction in an organizational context and developing a measure to assess the construct, our work supports a better understanding of the complex phenomenon of online interactions in organizations. First, this research transfers the concept of “online interaction” from the fields of marketing and teaching to an organizational context. This allows the phenomena to be described using a familiar concept. Online interactions provide new kinds of social relationships, and new ways of relating to others and to oneself in an organization. This research focuses more attention on employees' perceptions and experience during online interaction, which differs from previous research focused on EMSP and face to face interaction (Totterdell et al., 2012; Leonardi and Vaast, 2017). Further, the research echoed the view of Thompson (2018), finding that using communication media involves the creation of new forms of action and interaction, beyond ICT.

Second, this research applied both qualitative and quantitative methods to explore the dimensions of online interaction in an organizational context from the perspective of perception. We found that the conceptualization of the online interaction in an organizational context reflected two subscales: human–human interaction, human–computer interaction. The result expands and enriches online interaction research. Online interaction has received increasing discussion and attention in recent years; however, previous studies have mainly used scales derived from simple qualitative analysis and modifications (Qiao, 2019). Our study explored and analyzed the structure of online interaction using a root coding program, ensuring that the assessed structure of online interaction was consistent with reality. First, the study developed online interaction scales with 18 items having good reliability and validity. Organizations can use this type of instrument to assess levels of online interaction at workplaces, and then promote and utilize that interaction. In addition, the structural exploration and scale development build on views from previous online interaction studies and have some innovative findings. For example, this research confirms the dimensions of usefulness and ease of use described in previous online interaction studies (Gefen et al., 2003; Ahn et al., 2004), and also identifies a dimension that is more consistent with the psychological state of human–computer interaction in an organizational context: applicability. The results enrich research related to online interaction and may encourage researchers to focus attention on understanding human–computer interaction in the organizational context.

Finally, our findings corroborate widespread advice related to online work (Darling-Aduana, 2021), especially during COVID-19 (Shendell et al., 2021). The COVID-19 outbreak has reshaped people's attitudes, behaviors and values and has promote online interaction (Rydell and Kucera, 2021; Watson and Popescu, 2021). It is necessary to consider a new practice for working and communicating in organization to adapt to the COVID-19 epidemic. Our study may help employees adjust to this change in the organization and help prompt changes in organizational cultures that have not yet addressed the norms of online interaction, and the management of employee behavior in virtual communities.

## Practical Implications

This study also has several practical implications. First, for the individual, improving performance is not just important to the organization—it also provides a solid foundation for personal career development. The new online interaction scale developed for this study enables employee to gain insights into their own mental state and perceptions in a virtual environment and make necessary adjustments to improve them, which provide a new way for employees to improve their performance.

From the organization's perspective, online interaction can provide both an overall picture of employees' psychology and behavior in the virtual environment, and a more nuanced examination of where organizational digital management excels and where it may need improvement. ESMP are thriving in the workplace, but they come with risks. For example, employees can experience social media burnout, low efficiency, and poor quality when interacting online. Understanding the online interaction on ESMP may be an effective way for managers to gain insights to further optimize ESMP for work, and create a good environment and improve the quality of online interaction.

Finally, from a societal perspective, this research is helpful for advancing the digital management level of Chinese enterprises and conforms to the requirements proposed at the 2020 Central Economic Work Conference, which advised vigorously developing the digital economy. With investments in new infrastructure and the upsurge in the digital economy, many enterprises are embarking on a digital transformation. In particular, during the COVID-19 pandemic, a solid digital foundation has ensured organizational productivity, and digital management capabilities have gradually become a new driving force for organizational development. By studying online interaction in organizational contexts, this research provides a reference for enterprises to improve the level of digital management, and provides practical guidance for enterprises to promote the digital transformation of China's economy.

## Limitations and Future Research Recommendations

This study contains limitations that offer opportunities for future research. First, we did not verify predictive validity of online interactions. Future studies should consider important outcomes variables, such as physical health, behaviors, and organizational performance (Cunningham, 2021; Galbraith and Podhorska, 2021). In addition, future studies can refer to relevant research in the field of marketing (Andronie et al., 2021), and consider cognitive algorithmic processes and behavioral choices as regards online interactions in organizational contexts.

Second, experiences themselves are dynamic in nature. Recognizing this fact, researchers should look beyond the description of experience as a snapshot, and focus on the fluctuations in employees' feelings, perceptions, and behaviors (Wang et al., 2013; Zheng et al., 2015). To meet this goal, future research could further explore the online interaction fluctuations across different times of the day, using the scale developed in this study. This could capture the average state, and fluctuations, in online interactions.

In addition, it is an important limitation of this study that the scale was developed in a specific cultural context (China). The limitations of the current study call for researchers to collect further evidence of the reliability and validity from other cultural settings (e.g., Europe, USA, Africa, etc.).

Despite the limitations summarized here, this study adds value to the broader field. First, we applied a qualitative method (including theoretical deduction, interview, and expert evaluations) to identify and clarify the structural dimensions of online interaction in organizational situations. We also used multiple quantitative studies to validate this scale. Second, we developed a clear structure of online interaction in an organizational context. In this way, our study enriches the connotations associated with online interaction. We hope this study will stimulate greater interest in online interaction research, with the goal of creating a comprehensive, integrative understanding of this important topic.

## Data Availability Statement

The original contributions presented in the study are included in the article/supplementary material, further inquiries can be directed to the corresponding author/s.

## Author Contributions

XL was engaged in the investigation, literature search, and wrote the article. ZC was involved in contribution to the original draft and contribution to the last version. CZ involved in investigation and analyzed the data. QW involved in investigation and contribution to the last version. All authors contributed to the article and approved the submitted version.

## Funding

This research was funded by the Research Start-up Fund of WHPU and the Research and Innovation Initiatives of WHPU.

## Conflict of Interest

The authors declare that the research was conducted in the absence of any commercial or financial relationships that could be construed as a potential conflict of interest.

## Publisher's Note

All claims expressed in this article are solely those of the authors and do not necessarily represent those of their affiliated organizations, or those of the publisher, the editors and the reviewers. Any product that may be evaluated in this article, or claim that may be made by its manufacturer, is not guaranteed or endorsed by the publisher.
